# *“Everything Is Gonna Be Alright with Me”*: The Role of Self-Compassion, Affect, and Coping in Negative Emotional Symptoms during Coronavirus Quarantine

**DOI:** 10.3390/ijerph18042017

**Published:** 2021-02-19

**Authors:** Ana Filipa Beato, Leonor Pereira da Costa, Rita Nogueira

**Affiliations:** 1 HEI-Lab, Lusófona University, 1749-024 Lisbon, Portugal; leonor.costa@ulusofona.pt; 2 Clinical Center of Development PIN-Em Todas as Fases da Vida, 2770-022 Paço de Arcos, Portugal; rita.nogueira@pin.com.pt

**Keywords:** self-compassion, affect, coping, negative symptoms, coronavirus

## Abstract

Self-compassion has been associated with less distress, particularly when people face stressful and negative events. This study analyzed the mediation role of coping and affect in the relation between self-compassion and negative emotional symptoms during the quarantine decreed by Portuguese Health Authorities in the first phase of the coronavirus outbreak. A total of 428 Portuguese adults (75% women; *M*_age_ = 40.8, *SD* = 11.6) completed an online survey comprised by the Self-Compassion Scale (predictor); Short Version of Depression, Anxiety and Stress Scale (outcomes); The Positive and Negative Affect Schedule; and Brief-COPE. These instruments were adapted to COVID 19’s epidemic. Parallel mediation analyses demonstrated that self-compassionate participants were at less risk of suffering from symptoms of depression, anxiety, and stress during the quarantine. Plus, the relation between self-compassion and depressive, anxious, and stress symptoms were mediated by negative affect and dysfunctional coping style, but only for symptoms of depression. The findings support coping strategies and affect as links between self-compassion and distress but also the importance of separately analyzing the role of self-compassion, negative affect, and coping on symptoms of anxiety, depression, and stress. Low self-compassion might increase negative affect, maintaining stress responses to face demanding events during the COVID-19 epidemic. Results were discussed in the context of the pandemic outbreak.

## 1. Introduction

The pandemic of the novel coronavirus, COVID-19, brought an unprecedented time. The outbreak emerged in China, in the last months of 2019, and rapidly spread across the world [1]. The threat of a highly contagious virus with serious respiratory consequences forced national and international governments to take restrictive measures to prevent the contagion and limit the outbreak [2,3]. China, Italy, and England are examples of the countries most affected who adopted lockdown measures [4,5]. In turn, Portugal had declared a state of emergency on 18 March 2020, and strict quarantine was implemented [6]. In addition to the impacts at economic and political levels, many aspects of people’s lives have been significantly altered [7,8]. Daily activities, routines, and livelihoods were modified, and most of the population stayed at home, socially isolated themselves, and adopted transmission-related behaviors including wearing masks and health equipment. This means that everybody dealt with a lot of changes in their family, professional, and social contexts [9].

Previous research has revealed the significant impact of public health emergencies in mental health [10]. Symptoms of stress, anxiety, and depression are highly reported (e.g., [11,12]) not only because of the direct consequences of the disease, for example, the fear of becoming infected [13,14], but also because of the measures to contain the virus [15] such as social isolation [16]. So, once the adaptation of the population was challenged, there are crucial issues of mental health to consider in times of uncertainty and change during the outbreak of COVID-19.

The first studies already published help to understand the levels of psychological impact in stress emotional response [17]. In the initial stage of COVID-19, a study conducted in China analyzed the online posts from the most popular social network, Weibo, revealing an increase in negative emotions such as anxiety, depression, and indignation, and a decrease in positive emotions [18]. Another research group conducted an online survey to examine the psychological impact and mental health status in 1210 participants from different cities in China. The results show that 53.8% of respondents rated the psychological impact of the outbreak as moderate or severe, 16.5% reported moderate to severe depressive symptoms, 28.8% reported moderate to severe anxiety symptoms, and 8.1% reported moderate to severe stress levels [19]. As long as the number of confirmed and suspected cases and deaths related to COVID-19 infection have continued to escalate all over the world, the uncertainty and fear of being infected raised anxiety levels in healthy individuals as well in individuals with pre-existing mental health conditions [9]. Additionally, sleep disturbance [16], distress in healthy individuals [20], and social rejection, and discrimination [9] among the infected individuals have also been reported.

COVID-19 demands a stress-coping-adjustment process [19]—on the one hand, because that may affect physical health, on the other, because it affects mental health and well-being as well. Although the uncertainty of the pandemic circumstances may stimulate a natural and adaptive level of fear that enables the health-compliant behaviors [12,21,22], it also can precipitate new psychiatric symptoms in people without mental illness or aggravate the condition of those with pre-existing mental illness [23,24]. Added to the fear of contracting the virus, the significant changes in lives’ realities (working from home, home-schooling for children, lack of physical contact with others) contribute to the challenges and demands that people are facing during COVID-19. In this sense, they probably act to manage and react in ways that reduce the negative impact of this stressful event.

As the epidemic is ongoing, investigations and theoretical perspectives are being developed on how people are psychologically affected by and coping with the COVID-19 emergency. While the first and preliminary studies reported the psychological impacts, it is of interest to understand the process and factors that improve the functioning and well-being of individuals in this outbreak.

One of the constructs currently highlighted in the literature that may play an important role in how people deal with this potentially stressful event is self-compassion. Neff [25] introduces this concept as an attitude of being kind and nonjudgmental to one’s suffering. This involves three components: self-kindness, common humanity, and mindfulness. Self-kindness refers to an understanding of oneself rather than harshly judgmental and self-critical. Common humanity involves seeing one’s experiences as part of the human condition rather than as separating and isolating. Lastly, mindfulness involves awareness and acceptance of painful thoughts and feelings, rather than over-identifying with them.

In this sense, when facing negative events, such as COVID-19, where people are confronted with change and several challenges that could be painful or difficult to bear, instead of being critical and unkind, self-compassion allows relating self-to-self with the same care, tolerance, and concern as we treat significant others who are experiencing difficulties. Although there are no studies that associate this construct to the epidemic scenarios, there is much evidence that supports self-compassion in reducing distress and enhancing well-being [26] as an appealing active, approach-oriented view of emotion regulation when facing stressful events [27].

Several studies have found the negative association between self-compassion and negative affect, and also the positive correlation between self-compassion and positive affect [28,29], induced by imagined and remembered events [30]. Nevertheless, a lack of self-compassion is related to increased vulnerability to indicators of psychopathology. Self-criticism, negative self-evaluation, shame, submissive behavior, rumination, and worry showed to be significantly and negatively correlated with self-compassion [31,32,33]. Another robust finding points out that greater self-compassion is consistently related to less depression and anxiety [25,30], and it has a significant direct effect after controlling the mediators such as those mentioned above [32]. It seems as though this growing evidence suggests this construct as a protective factor to the promotion of emotional resilience [34], even of Post-Traumatic Stress Disorder [35]. Self-compassion presents as a promising buffer that moderates emotional reactivity to negative events, mitigating the negative impact of unproductive repetitive thinking in depression and anxiety [32].

According to Neff [25], it can be viewed as a useful emotional approach coping strategy where uncomfortable or distressing feelings are not avoided but enables clear appraisal to the immediate situation and consequently the adoption of actions that change oneself and or the environment in appropriate and effective ways. The inevitable pain and discomfort are not amplified or perpetuated by self-attacking thoughts, feelings of isolation, or over-identification. They are tolerated with self-kindness, understanding, balance perspective [36], and a sense of shared humanity.

Thus, it is important to distinguish self-compassion from apathy or laziness [30]. As clarified by Neff [37], self-compassion, in contrast, involves the motivation and personal initiative to make needed changes in one’s life and modify unproductive behaviors [25]. This led individuals to choose powerful actions to take under certain circumstances and react to negative events with a chance to grow. For example, Neff and colleagues [38] verified that self-compassion is positively related to emotion-focused coping strategies of acceptance and positive reinterpretation/growth and negatively related to a focus on negative emotions and avoidance-oriented coping. Regarding problem-solving as noted by Allen and Leary [39], there is no consistency in reported results. These authors indicated that self-compassion and problem-solving may be linked by the perception of control. If there is a lower perception of control, self-compassionate individuals will not engage in actions to fix it; they will focus on emotional coping strategies. Furthermore, self-compassion is consistently linked with adaptive coping and positive cognitive reactions such as optimism and perspective-taking [36,40,41]. As verified by Lloyd and colleagues [42], dysfunctional strategies mediated the relationship between self-compassion and distress. So, higher levels of self-compassion reduce the likelihood of dysfunctional strategies, protecting individuals from distress. This fact is important because it allows glimpsing the relationship that is very documented between higher self-compassion and less distress in the face of stressful events, suggesting that one mechanism by which self-compassion may act is through unviable dysfunctional coping strategies. Current research on well-being indicates that self-compassion is associated with life satisfaction, happiness, optimism [43], and subjective well-being [38].

In summary, self-compassion can be conceptualized as a self-related attitude that promotes well-being, positive functioning, and recovery after experiencing a stressor. Therefore, self-compassion can give clues to explore stress management in terms of how individuals regulate their emotions and their reactions to external events, such as all the direct and indirect consequences of COVID-19. We can highlight two possible mechanisms involved between self-compassion and less distress. On one hand, adopting a self-critic critical cognitive appraisal (that is, blaming the self) might often signal higher event stress and escalating negative affect [44]. On the other hand, blaming the self and getting stuck in suffering usually triggers avoidant coping, which is recognized as a maladaptive response to a variety of stressors, and there are important risk factors for this because they increase the severity and duration of the stressors as well as the negative psychological symptoms and distress [45]. Thus, this investigation proposes to analyze the mediation role of coping and affect in the relation between self-compassion and depression, anxiety, and stress, separately, during the quarantine decreed by Portuguese Health Authorities in the first phase of the coronavirus outbreak. Based on the previous revised literature, we hypothesized that (H1) Self-compassion is negatively associated with negative emotional symptoms (depression, anxiety, stress); (H2) Self-compassion is positively related to positive affect and, inversely, to negative affect; (H3) Self-compassion is positively related to effective coping styles (i.e., problem-focused and emotion-focused) and negatively associated with dysfunctional coping; and (H4) Lower self-compassion predicts higher symptoms of depression, anxiety, and stress, through negative affect and dysfunctional coping.

## 2. Materials and Methods

### 2.1. Participants

The participants of this study consisted of 428 Portuguese adults (75% women; *M*_age_ = 40.8, *SD* = 11.6; ranging from 18 to 71 years old). Nearly 65% of respondents self-identified as middle class as their socio-economic status, 18.3% self-identified as lower or lower-middle class, and 17% self-identified as upper-middle or upper classes. More than 54% were married or lived together with their partners, 18.6% were single, 17.5% were engaged, and 9.4% had another relationship status. Almost all the participants possess Portuguese nationality (97,9%). Furthermore, 72.9% had a college degree, 15.9% completed the college degree, and 11.5% had lower education degrees. Nearly 81% were employed or are student workers, 6% were students, 5.5% were unemployed, and 9.2% were in other circumstances (e.g., retired, pensioners). During the mandatory quarantine, 67.7% of the respondents were isolated at home; most of them had full-time telework jobs (46%), while 28.3% were working out of their home. In addition, nearly 50% were caring for minors (41.4%), dependent adults (6.4%), or both (1.8%). Only 19.5% of them considered their workplaces moderate to high risk for COVID-19. Approximately 23% of the participants had relevant physical conditions, chronic diseases, and/or were pregnant, and have a current or have had a past psychological condition, i.e., they have had in the past or were having at the time of the questionnaire psychological/psychiatric support (39%). The study included adults living in Portugal during the mandatory quarantine due to COVID-19. Duplicate responses were excluded, as were participants living temporarily out of the country and blank responses that did not conclude the survey (*n* = 5). In addition, the participants who presented at least one missing value in the main variables or co-variables were excluded from the final sample (*n* = 7).

### 2.2. Measures

#### 2.2.1. Self-Compassion

To measure the thoughts, emotions, and behaviors associated with the three components of self-compassion, *Self-compassion Scale* was used (SELFCS [46]; Portuguese version: Gouveia and Castilho, 2006). The 26-item scale asks people to identify how often they respond to feelings of inadequacy and/or suffering in difficult moments. In this case, we adjusted the instructions to evaluate how the participants responded specifically to difficult times caused by the epidemic. The instrument has six dimensions and has good internal consistency in this study: Self-kindness (α = 0.87; “I try to be loving toward myself when I’m feeling emotional pain”), Self-Judgment (α = 0.86, “I’m disapproving and judgmental about my own flaws and inadequacies”), Common Humanity (α = 0.81, “When things are going badly for me, I see the difficulties as part of life that everyone goes through.”), Isolation (α = 0.78, “When I think about my inadequacies, it tends to make me feel more separate and cut off from the rest of the world”), Mindfulness (α = 0.84, “When something upsets me, I try to balance my emotions”), and Over-identified (α = 0.84, “When I’m feeling down I tend to obsess and fixate on everything that’s wrong”). Subscale scores are computed by calculating the mean of subscale item responses. To test our hypotheses, we used the overall score, representing the mean of subscale item responses (SCS; α = 0.91). Higher scores denote more compassionate responses.

#### 2.2.2. Affect

Perceptions of positive and negative affect were obtained using the short version of *The Positive and Negative Affect Schedule* (PANAS; [47]; Portuguese version; [48]) is a 10-item self-report scale that has two columns with five adjectives, which participants use to rate positive (e.g., determined) and another five adjectives to rate negative emotions (e.g., distressed). Respondents were instructed to select the emotions they have felt in the last two weeks, concerning the specific period of the quarantine, using a rating scale of 1 (very slightly or not at all) to 5 (extremely). The instructions were adapted to the emotions that occurred in the last weeks, during the quarantine. Items are summed and scored in a range from 10 to 50. Higher scores reveal more levels of positive or negative affect. Adequate internal consistency was achieved for both positive affect (α = 0.84) and negative affect (α = 0.88) scales.

#### 2.2.3. Coping Styles

The *Brief-COPE* [49]; Portuguese version; [50]) is a 28-item self-report questionnaire that was adapted for the purpose of our study and that allowed people to identify how often they used various effective and ineffective strategies to cope with hardships during the pandemic. The response format was a Likert scale ranging from 0 (*I haven’t been doing this at all*) and 3 (*I’ve been doing this a lot*) and has 14 subscales, which might be agglomerated to determine primary two coping styles: Avoidant and Approach coping. In our study, we followed prior studies (e.g., [51] using three types of coping strategies, to understand the differences between the effective coping styles. Dysfunctional coping includes the subscales “Self-distraction”, “Denial”, “Substance use”, “Behavioral disengagement”, “Venting” and “Self-blame”; emotion-focused coping that includes the subscales “Use of emotional support”, “Positive reframing”, “Humor”, “Acceptance”, “Religion”; and problem-focused coping that includes the subscales “Active coping”, “Use of instrumental support” and “Planning”. The Cronbach’s alphas were adequate for dysfunctional (α = 0.68), problem-focused (α = 0.79), and emotion-focused (α = 0.76) coping styles. We also calculated alphas for each specific strategy. They all were above 0.60 except self-distraction, which presented lesser adequate internal consistency (α = 0.53), and self-blaming (α = 0.53).

#### 2.2.4. Negative Emotional Symptoms

The Short Version of Depression, Anxiety and Stress Scale ([52]; Portuguese version; [53]) was used to assess the degree of severity of core symptoms of depression, such as dysphoria, hopelessness, devaluation of life, self-deprecation, lack of interest/involvement, anhedonia, and inertia (e.g., “I was unable to become enthusiastic about anything”); anxiety, namely autonomic arousal, skeletal muscle effects, situational anxiety, and subjective experience of anxious affect (e.g., “I felt scared for no reason”); and stress, such as difficulty relaxing, nervous arousal, and being easily upset, agitated, irritable, over-reactive and impatient (e.g., “I found it difficult to relax”). In line with the other instruments, the instructions were adapted in order to assess symptoms that occurred during the quarantine. This test consists of a list of 21 symptoms, each of which is rated on a four-point Likert scale on how much they have experienced that symptom during the preceding weeks (0 = Did not apply to me at all; 1 = Applied to me to some degree or some of the time; 2 = Applied to me to a considerable degree or a good part of the time; 3 = Applied to me very much, or most of the time). Results are summed for each dimension, and scores vary between 0 and 42. Higher scores indicate greater emotional symptoms. The average results range from 0 to 9. Good internal consistency was acquired for our study in all subscales (symptoms of depression: α = 0.87, anxiety α = 0.82, stress: α = 0.90).

### 2.3. Procedures

All procedures in this study were approved by the Ethics and Deontology Committee of Psychology and Life Sciences School of University Lusófona. The participants were recruited through a non-probability sampling method (convenience sampling). Interested participants completed an online survey hosted by Google Forms^®^ (Online survey services) containing instructions, informed consent, and five questionnaires. All participants confirmed their willingness to participate after they read the Informed Consent, which had information regarding the aims of the study, the voluntary nature of their participation, and all the security procedures researchers underwent to guarantee confidentiality (e.g., elimination of ID number and geolocations from questionnaires before the analyses).

Data collection occurred from 10 April until 4 May 2020. This period correspond to the mandatory COVID-19 quarantine declared by Portuguese Authorities (from 19 March until 4 May 2021). Only “essential” workers and volunteers could continue to work outside of their homes.

### 2.4. Data Analyses

Statistical assumptions and Pearson’s correlation coefficients were analyzed using SPSS (v. 26; IBM Corporation, Armonk, NY, USA). To test our hypotheses and examine the effect of Self-Compassion on Depression, Anxiety, and Stress, three parallel mediation models were conducted using PROCESS version 3.3 for IBM SPSS Statistics (Model 4; [54]), with affect and coping strategies as mediators. To test the indirect effects, we used 5000 bootstrap samples to generate the percentile bootstrap confidence intervals. Initial exploration of our data suggested that several socio-demographic variables should be controlled. Thus, gender (0 = Female; 1 = Male), age, education level (1 = Primary Education to 7 = Doctoral Studies), problematic physical conditions (0 = No; 1 = Yes), being in quarantine (0 = No; 1 = Yes), psychological conditions (0 = Never had psychological support, 1 = Already had psychological support sometime in my life, and caring for dependent people during quarantine (0 = No; 1 = Yes) were used as covariates in all subsequent analyses.

## 3. Results

To confirm our first hypothesis on the relation between self-compassion and affect, coping, and negative emotional symptoms, zero-order correlations were performed. These results and descriptive statistics of the variables are presented in Table 1. Preliminary analyses of our data reveal, on average, that the participants of the study are at normal levels of symptoms of depression, anxiety, and stress [53]. Self-compassion illustrated a mean score above the midpoint of the scale (*t* (434) = 26.34, *p* < 0.001). Positive affect exhibited a mean score above the midpoint of the scale (*t* (435) = 10.39, *p* < 0.001) and negative affect was significantly below the midpoint of the scale (*t* (435) = −9.78, *p* < 0.001). Mean scores of participants’ emotion-focused (*t* (434) = 4.19, *p* < 0.001) and problem-focused (*t* (434) = 9.97, *p* < 0.001) coping strategies were both above the midpoint of the scale, while dysfunctional coping mean score was below the midpoint of the scale (*t* (434) = −36.68, *p* < 0.001).

There were three models tested where self-compassion was entered as the predictor, positive affect, negative affect, and the three types of coping strategies (emotional, problem-oriented, and dysfunctional) as parallel mediators, and symptoms of depression (Model 1), anxiety (Model 2), and stress (Model 3) as outcomes.

**Figure 1:**
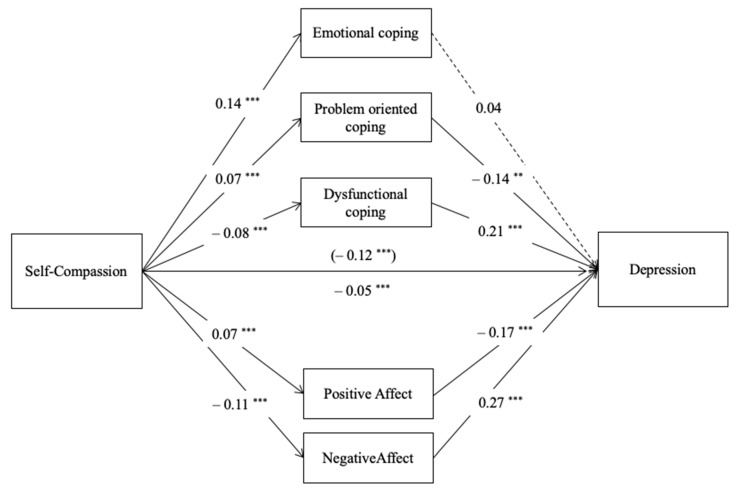
Parallel mediation model with path coefficients (unstandardized): self-compassion, positive affect, negative affect, emotional coping, problem-oriented coping, dysfunctional coping, and symptoms of depression (M1). Note: ** *p* < 0.01 *** *p* < 0.001, *c’* (direct effect) above the line and *c* (total effect) below the line, dashed lines are not-significant paths.

Model 1 explained 53.8% of the variance of the variable depression, which was significant (R^2^ = 0.538, *F* (13,414) = 37.11, *p* < 0.001); see Figure 1. Results showed a negative total effect of self-compassion on symptoms of depression (*b* = −0.118, *SE* = 0.009, *t* = −12.56, *p* < 0.001) that remained negative and significant when accounting for all the mediators (*b* = −0.053, SE = 0.010, *t* = −5.13, *p* < 0.001). Self-compassion positively predicted positive affect (*p* < 0.001) and the engagement in emotional and problem-oriented coping strategies (always, *p* < 0.001), while negatively predicted negative affect (*p* < 0.001) and dysfunctional coping strategies (*p* < 0.001). Negative affect and dysfunctional coping positively predicted symptoms of depression (always, *p* < 0.001) while positive affect (*p* < 0.001) and problem-focused coping (*p* = 0.009) negatively predicted symptoms of depression (*p* = 0.009). Findings of this model demonstrated a significant indirect effect of self-compassion on symptoms of depression through positive affect (*b* = −0.012, *BootSE* = 0.004, *Boot CI 95%* [−0.020, −0.006]), negative affect (*b* = −0.029, *BootSE* = 0.006, *Boot CI 95%* [−0.043, −0.018]), dysfunctional coping strategies (*b* = −0.018, *BootSE* = 0.005, *Boot CI 95%* [−0.028, −0.010]) and through problem-oriented coping strategies (*b* = −0.010, *BootSE* = 0.004, *BootCI 95%* [−0.019, −0.002], yet not through emotional coping strategies (*b* = 0.005, *SE* = 0.005, *Boot CI 95%* [−0.004, 0.015]. Detailed statistical results can be found in Table S1 in the Supplementary Materials.

**Figure 2:**
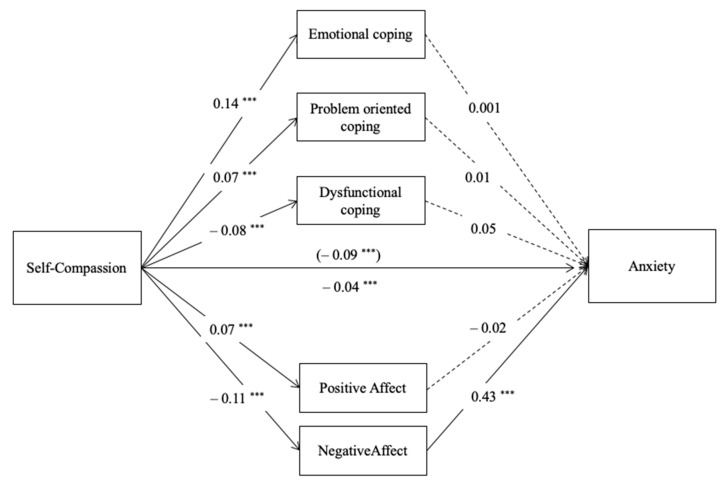
Parallel mediation model with path coefficients (unstandardized): self-compassion, positive affect, negative affect, emotional coping, problem-oriented coping, dysfunctional coping, and anxiety (M2). Note: *** *p* < 0.001, *c’* (direct effect) above the line and *c* (total effect) below the line, dashed lines are not-significant paths.

Model 2 explained 55.6% of the variance of anxiety, which was a significant model (R^2^ = 0.556, *F* (13,414) = 39.87, *p* < 0.001), see Figure 2. The total effect of self-compassion on anxiety was negative and significant (*b* = −0.09, *SE* = 0.01, *t* = −9.91, *p* < 0.001), and this became weaker, while remaining negative, when the mediators were accounted for (*b* = −0.04, *SE* = 0.01, *t* = −4.03, *p* = 0.001). The results of this model show that there was only one significant indirect effect of self-compassion on symptoms of depression through negative affect (*b* = −0.05, *BootSE* = 0.007, *Boot CI 95%* [−0.062, −0.034]). None of the three types of coping strategies considered were predictors of anxiety (*p* = 0.97, *p* = 0.80 and *p* = 0.19, for emotional, problem-oriented and dysfunctional coping, respectively), and positive affect also did not predict anxiety (*p* = 0.57). None of these variables showed to mediate the negative relation between self-compassion and anxiety. Detailed statistical results can be found in Table S2 in the Supplementary Materials.

**Figure 3:**
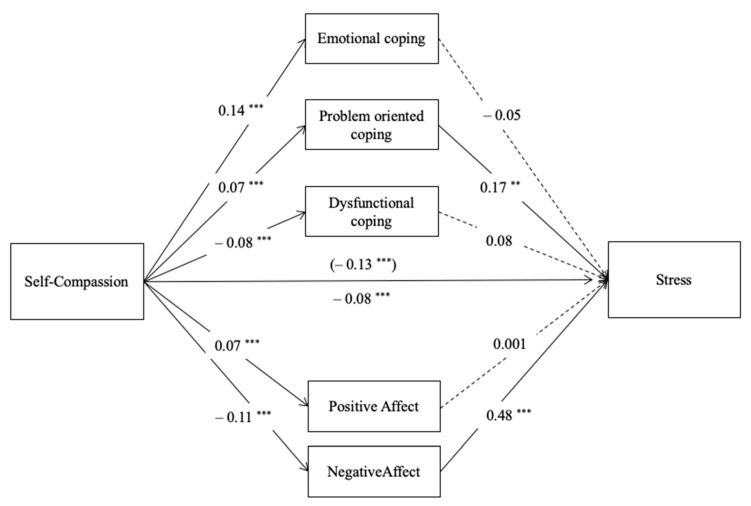
Parallel mediation model with path coefficients (unstandardized): self-compassion, positive affect, negative affect, emotional coping, problem oriented coping, dysfunctional coping and stress (M3). Note: ** *p* < 0.01, *** *p* < 0.001, *c’* (direct effect) above the line and *c* (total effect) below the line, dashed lines are not-significant paths.

Model 3 explained 59.1% of the variance of the variable stress and was significant (R^2^ = 0.591, *F* (13,414) = 46.01, *p* < 0.001); see Figure 3. As previous outcomes, negative affect predicted stress (*p* < 0.001). Moreover, problem-oriented coping strategies were positively related to stress (*b* = 0.17, *SE* = 0.06, *p* = 0.005), and dysfunctional coping was only marginally related to stress (*b* = 0.08, *SE* = 0.04, *p* = 0.07). Emotional coping was not significantly related to stress (*p* = 0.23). The results reflect a significant indirect effect of self-compassion on stress through negative affect (*b* = −0.05, *BootSE* = 0.008, *Boot CI 95%* [−0.068, −0.037]) and through problem-oriented coping strategies (*b* = 0.01, *BootSE* = 0.005, *Boot CI 95%* [0.004, 0.023]). Detailed statistical results can be found in Table S3 in the Supplementary Materials.

**Figure 4:**
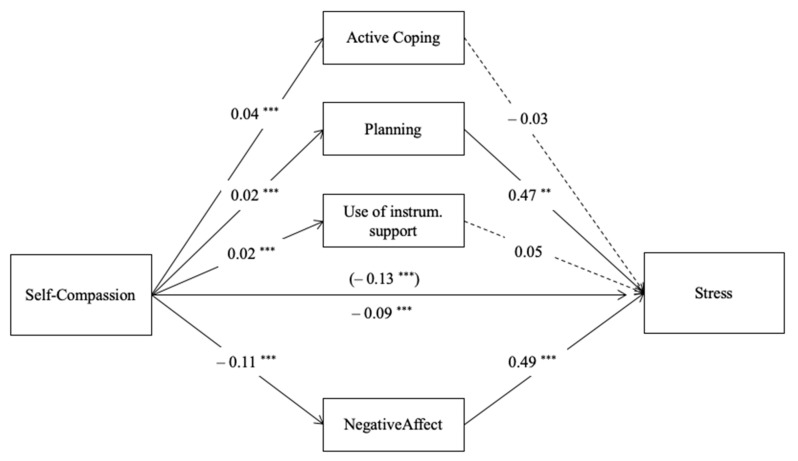
Parallel mediation model with path coefficients (unstandardized): self-compassion, negative affect, active coping, planning, use of instrumental support coping and stress. Note: ** *p* < 0.01, *** *p* < 0.001, *c’* (direct effect) above the line and *c* (total effect) below the line, dashed lines are not-significant paths.

To further address the unexpected result regarding the mediating effect of self-compassion on stress through problem-oriented coping strategies, we explored the mediating role of each component of the problem-oriented coping strategies (active coping, planning, and seeking instrumental support) on the relation between self-compassion and stress. For that, we conducted another parallel mediation analysis (Model 4; [55]) considering the mediators that resulted in significant (negative affect) and substituting the problem-oriented coping by its three components (see Figure 4), controlling for the same covariates in the previous models. Zero-order correlations of the variables presented in this Model 4 are presented in Table 2.

This model was significant (R^2^ = 0.591, *F* (12.416) = 50.23, *p* < 0.001) and explained 59.1% of the variance of the variable stress. The results showed that negative affect remained as a positive predictor of stress (*p* < 0.001) and a mediator of the relation between self-compassion and stress (*b* = 0.05, *BootSE* = 0.008, *Boot CI 95%* [−0.070, −0.038]). Regarding the three specific coping strategies within the general problem-focused coping strategy, findings of this model revealed that only the “planning” coping strategy is a positive predictor of stress (*p* = 0.002) and showed to be a significant mediator of the relation between self-compassion and stress (*b* = 0.01, *BootSE* = 0.004, *Boot CI 95%* [0.004, 0.019]). In contrast, the link between self-compassion and stress was not explained by the “active coping” strategy (*b* = −0.001, *BootSE* = 0.006, *Boot CI 95%* [−0.011, 0.009]) and “seeking of instrumental support” strategy (*b* = 0.001, *BootSE* = 0.002, *Boot CI 95%* [−0.002, 004]). Detailed statistical results can be found in Table S4 in the Supplementary Materials.

## 4. Discussion

The present study aimed to explore the relationship between self-compassion, affect, coping styles, and negative emotional symptoms in a sample of Portuguese adults during the novel coronavirus quarantine. As predicted by our first hypothesis, self-compassion was negatively associated with symptoms of depression, anxiety, and stress. In line with previous studies and meta-analyses (e.g., [56], our results have shown that self-compassionate people present better mental health; that is, they are at less risk to suffer from symptoms of depression, anxiety, and stress during stressful life events, such as those brought on by the coronavirus outbreak.

Not as a surprise, our second hypotheses were also confirmed. Self-compassionate people tend to feel less negative affect and higher positive affect. This result might reflect mindful self-compassion. Being aware, observing their thoughts and feelings in a nonjudgmental manner, might help people accept negative emotions, rather than avoid or change them, decreasing the feelings of over-identification and of being overwhelmed by negative affect. This tendency to neutralize and accept negative emotions as part of the human experience might help people regulate these feelings and, as a plus, be open to more positive affect [38].

Results also confirmed the third hypothesis. As in various studies reviewed by Allen and Leary [39], self-compassionate people tend to engage in fewer dysfunctional coping strategies and in more adaptive ones. The literature has not yet specified which strategies are more associated with self-compassion, reflecting mixed results for the association between self-compassion and problem-focused coping. Our study added some relevant elements to the field by indicating that when we consider broader styles, self-compassionate people seem to adopt both emotional-focused and problem-focused coping styles, and less dysfunctional coping to deal with the quarantine stressors. These results are consistent with previous studies [38,39,57], demonstrating that self-compassion helps individuals adopt greater adaptive coping strategies (e.g., positive reframing). According to a recent study from Chishima and colleagues [36], self-compassion promotes adaptive coping through a reduced threat perception and increased controllability over stressful events. In this sense, future studies should clarify the connections between self-compassion and (in)adaptive coping in the context of the coronavirus, namely the role of threat perceptions and sense of control.

The fourth hypothesis of our study intended to explore if negative affect and dysfunctional coping mediated the relation between self-compassion and the different negative emotional symptoms considered. This hypothesis was partially confirmed; that is, only negative affect consistently mediated this association, although different patterns of results were found across the three outcomes. The results show that adopting an increased self-compassionate perspective during the quarantine allowed a reduction of negative emotions and, as a result, these individuals were at lesser risk of feeling depressed, anxious, and/or stressed out. This finding was in agreement with antecedent studies, confirming a protective role of self-compassion by increasing positive automatic thoughts, approaching the situation with mindfulness, promoting positive mindsets, as positive affect [29,43,58], and in line with Gilbert´s theoretical model [59] that posits that self-compassion may activate parasympathetic activity and down-regulate sympathetic activity, reducing negative mind states, such as negative affect. Consequently, this path decreases the risk for symptoms of anxiety, stress, and depression [26,60]. In contrast, people who possess low self-compassion tend to struggle with their negative emotions and remain hooked to them, increasing the probability of negative symptoms to arise or maintain pre-existing psychological problems.

Contrarily to our expectation, dysfunctional coping only played a role in the relation between self-compassion and symptoms of depression. This result gives us further clues to understand the pathways through which a lack of self-compassion may lead to or increase depressive symptoms. As found in preliminary studies, less compassionate individuals tend to be more cognitively and behaviorally avoidant and engage in ruminative strategies, which reinforces depressive symptoms [57]. Unexpectedly, self-compassionate individuals tended to adopt more problem-focused coping strategies, which, in turn, put them at greater risk for stress. To clarify this result, we performed a post-hoc mediation analysis with the specific strategies of problem-solving coping style and negative affect as mediators. Only planning increased stress responses in self-compassionate individuals. Although, as shown in previous studies, self-compassionate people tend to use more proactive coping mechanisms, such as making plans to deal with or to prevent problems [61]. We hypothesize that in the context of the first phase of the pandemic caused by the novel coronavirus, planning might have been ineffective to reduce stress and might even aggravate it. The adverse effect of this strategy might be due to the uncertainty and unstable evolution of the epidemic. The global COVID-19 pandemic has resulted in psychological chaos, uncontrollability, and constant uncertainty led by radical restrictions and recurring changes in individuals’ daily routines [62]. In this sense, individuals’ psychological well-being may fluctuate in their daily lives, impacting the way individuals cope with the disease and struggle to know how to plan for their future. Deciding which will be the next steps to solve problems and prevent issues seem to be a hard task to accomplish when individuals face global pandemics, given their lack of control and uncertainty on important issues, such as the evolution of the disease, its treatment, or its impact on health and socio-economy.

The present study has several limitations that must be highlighted. First, responses are potentially flawed by recall bias, motivation, accessibility to the survey, and social desirability. Second, respondents who completed the survey represent a self-selected group recruited in the community, presented medium negative emotional symptoms, and were not sufficiently heterogeneous. Third, coping styles were measured through a short version of general coping styles, which does not reflect the strategies and needs related directly to this pandemic’s stressors. Fourth, this cross-sectional study was conducted in the acute first phase of the quarantine and might have been influenced by the way the virus spread throughout Portugal, by government restrictions measures, and social impact influenced by social media and news.

For these reasons, future studies should include experimental and longitudinal designs to manipulate self-compassion and/or coping strategies and analyze their causal effect on psychological outcomes. They should also examine how self-compassion, affect, and coping evolve and predict better or worse emotional outcomes over time, namely in different phases of the pandemics. Moreover, future studies could use qualitative and/or mixed methods to improve and comprehend how people perceive COVID-19’s infection and its impact. Future studies could also examine which coping strategies are perceived as more effective or ineffective in dealing with distress, and how self-compassion mindful strategies might work best from person to person, according to their characteristics, stressors, and socio-cultural contexts. Lastly, this study could be replicated in different cultures, professions, ethnic and minority groups, and with clinical samples to explore if these results change across multiple settings and populations.

Despite these limitations, our findings give further evidence on how self-compassion is generally associated with less distress and more adaptive coping mechanisms during pandemic scenarios. In line with this, future longitudinal studies should clarify if self-compassion functions as a protective factor against psychopathology in these epidemic contexts. Moreover, our study contributes to understanding self-compassion, stress, and coping strategies, as we provide additional evidence on affect and coping as mediators and key factors to clarify the relation between self-compassion and distress during global pandemics. These findings are useful not only for researchers as well as for practitioners who engage in clinical interventions to improve clients’ mental health. Furthermore, our findings support that certain coping strategies that lessen distress in one situation or moment may be ineffective or even detrimental to the individual in another. Thus, more than defining if coping is good or bad, or effective or ineffective, one must well consider the many specificities of the context, moment, situation, and the goals of the individuals before drawing firm conclusions [63]. In addition, having delivered analyses with different outcomes, we conclude that depressive, anxious, and stress symptoms are maintained and are reinforced by different mechanisms, and they perhaps are caused by diverse risk factors, too. Thus, the question of disorder-specificity remains unsettled and requires further clarification in future investigations.

## 5. Conclusions

In addition to all the negative impacts of social isolation and restrictions derived from this pandemic (see [64], for a review), protective factors and preventive strategies are most important to be identified to better respond to this worldwide stressor and prepare mankind for future similar outbreaks. Mental health scientists highlighted the importance of longer-term strategies and coping mechanisms to deal adaptively with COVID-19 [65]. Self-compassion might well be one of the most promising individual resources to deal with the negative impact of COVID-19. In this sense, our study highlighted the role of improving and incorporating self-compassion mindful techniques in both clinical and community contexts. Professional interventions during the quarantine should provide empirically supported programs to deal with self-criticism or self-coldness and promote greater self-compassion attitudes (e.g., [66]). Furthermore, mobile [67] and web-based [68] mindful compassion interventions have shown to be effective and might be particularly helpful during epidemic confinements.

The positive affect and the adaptive coping styles associated with self-compassion evidenced the importance of promoting more self-kind, mindful, and accepting attitudes during epidemic outbreaks. Future research and interventions on self-compassion and coping should also address that pandemic adversities need specific response strategies to guarantee behavioral health needs of people from diverse developmental stages, as well as greater risk groups (e.g., health professionals, volunteers, people with prior physical and mental health conditions).

## Figures and Tables

**Table 1:** Descriptive statistics and correlations of the variables of interest for Models 1−3.

	*M*	*SD*	2	3	4	5	6	7	8	9
1. Self-Compassion	85.45	16.19	−0.553 ***	−0.469 ***	−0.517 ***	0.298 ***	−0.420 ***	0.407 ***	0.342 ***	−0.358 ***
2. Symptoms of Depression	3.60	3.72		0.635 ***	0.621 ***	−0.348 ***	0.541 ***	−0.164 **	−0.178 ***	0.477 ***
3. Anxiety	2.97	3.49			0.725 ***	−0.162 **	0.689 ***	−0.130 **	−0.058	0.385 ***
4. Stress	6.36	4.60				−0.128 **	0.678 ***	−0.109 *	0.022	0.439 ***
5. Positive Affect	14.56	4.13					−0.114 *	0.225 ***	0.323 ***	−0.110 *
6. Negative Affect	10.42	4.43						−0.153 **	−0.029	0.399 ***
7. Emotional Coping	16.03	5.13							0.671 ***	0.140 **
8. Problem Coping	10.66	3.47								0.223 ***
9. Dysfunct. Coping	10.66	4.17								-

* *p* < 0.05, ** *p* < 0.01, *** *p* < 0.001.

**Table 2:** Correlations of the variables of interest for Model 4.

	2	3	4	5	6
1. Self-Compassion	−0.517 ***	−0.420 ***	0.434 ***	0.300 ***	0.126 **
2. Stress		0.678 **	−0.123 *	0.042	0.107 *
3. Negative affect			−0.166 **	−0.017	0.084
4. Active coping				0.652 ***	0.344 ***
5. Planning					0.405 ***
6. Instrumental support					-

* *p* < 0.05, ** *p* < 0.01, *** *p* < 0.001.

## Data Availability

The data presented in this study are openly available in https://osf.io/59gs6/?view_only=e78daad636294c278d192a80f0e1285c (accessed on 19 February 2021).

## Supplementary Materials

The following are available online at https://www.mdpi.com/1660-4601/18/4/2017/s1, Table S1: Unstandardized Regression Coefficients, Standard Errors, and Model Summary Information for the Parallel Multiple Mediator Model 1, Considering Symptoms of Depression as the Dependent Variable, Table S2: Unstandardized Regression Coefficients, Standard Errors, and Model Summary Information for the Parallel Multiple Mediator Model 2, Considering Anxiety as the Dependent Variable, Table S3: Unstandardized Regression Coefficients, Standard Errors, and Model Summary Information for the Parallel Multiple Mediator Model 3, Considering Stress as the Dependent Variable, Table S4: Unstandardized Regression Coefficients, Standard Errors, and Model Summary Information for the Parallel Multiple Mediator Model 4, Considering Stress as the Dependent Variable and the Problem Oriented Coping Strategies and Negative Affect as Mediators.
