# Supplementary material for: “Everything Is Gonna Be Alright with Me”: The Role of Self-Compassion, Affect, and Coping in Negative Emotional Symptoms during Coronavirus Quarantine
Source: Int J Environ Res Public Health. 2021 Feb 19;18(4):2017. doi: 10.3390/ijerph18042017 (PMC7923103; doi:10.3390/ijerph18042017)
Supplement: Supplementary file 1 [file ijerph-18-02017-s001.pdf]

Table S1

Unstandardized Regression Coefficients, Standard Errors, and Model Summary Information for the Parallel Multiple Mediator Model 1, considering Symptoms of Depression as the Dependent Variable.

	Dependent Variables																				
	Emotional Coping (M1)			Problem Oriented Coping (M2)			Dysfunctional Coping (M3)			Positive Affect (M4)			Negative Affect (M5)			Depression (Y)			Depression (Y)		
Predictors	Coeff.	*SE*	*p*	Coeff.	*SE*	*p*	Coeff.	*SE*	*p*	Coeff.	*SE*	*p*	Coeff.	*SE*	*p*	Coeff.	*SE*	*p*	Coeff.	*SE*	*p*
Self-Comp. (X)	0.14	0.01	0.00	0.07	0.01	0.00	-0.08	0.01	0.00	0.07	0.01	0.00	-0.11	0.01	0.00	-0.12	0.01	0.00	-0.05	0.01	0.00
M1	-	-	-	-	-	-	-	-	-	-	-	-	-	-	-	-	-	-	0.04	0.04	0.27
M2	-	-	-	-	-	-	-	-	-	-	-	-	-	-	-	-	-	-	-0.14	0.05	0.01
M3	-	-	-	-	-	-	-	-	-	-	-	-	-	-	-	-	-	-	0.21	0.04	0.00
M4	-	-	-	-	-	-	-	-	-	-	-	-	-	-	-	-	-	-	-0.17	0.03	0.00
M5	-	-	-	-	-	-	-	-	-	-	-	-	-	-	-	-	-	-	0.27	0.03	0.00
Cov. 1	1.88	0.47	0.00	0.53	0.34	0.12	1.13	0.4	0.00	-0.33	0.41	0.42	0.57	0.4	0.15	0.89	0.32	0.00	0.44	0.28	0.12
Cov. 2	-0.85	0.46	0.07	-0.07	0.33	0.83	-0.15	0.39	0.70	-0.03	0.4	0.93	1.37	0.39	0.00	-0.44	0.31	0.16	-0.76	0.27	0.01
Cov. 3	-0.09	0.46	0.84	-0.04	0.33	0.90	0.41	0.38	0.29	0.43	0.4	0.28	0.64	0.39	0.1	0.52	0.31	0.09	0.34	0.26	0.20
Cov. 4	0.01	0.02	0.66	-0.01	0.01	0.39	-0.02	0.02	0.32	0.00	0.02	0.94	-0.03	0.02	0.13	-0.02	0.01	0.07	-0.02	0.01	0.15
Cov. 5	-1.67	0.52	0.00	-0.92	0.37	0.01	-1.21	0.44	0.01	0.62	0.45	0.17	-1.74	0.44	0.00	-0.82	0.35	0.02	-0.05	0.31	0.88
Cov. 6	-0.01	0.17	0.93	0.21	0.12	0.08	-0.26	0.14	0.06	0.38	0.14	0.01	-0.09	0.14	0.54	-0.24	0.11	0.03	-0.06	0.10	0.51
Cov. 7	-0.35	0.53	0.5	0.02	0.38	0.96	-0.46	0.45	0.31	0.05	0.46	0.91	0.99	0.45	0.03	0.71	0.36	0.05	0.56	0.31	0.07
Constant	4.35	1.62	0.01	3.84	1.16	0.00	19.64	1.37	0.00	6.53	1.41	0.00	20.16	1.38	0.00	15.39	1.09	0.00	7.31	1.28	0.00
	R^2 = 0.23			R^2 = 0.15			R^2 = 0.19			R^2 = 0.11			R^2 = 0.27			R^2 = 0.36			R^2 = 0.54		
	F(8,419) = 15.79, p <.001			F(8,419) = 9.19, p <.001			F(8,419) = 12.17, p <.001			F(8,419) = 6.63, p <.001			F(8,419) = 19.60, p <.001			F(8,419) = 29.63, p <.001			F(13,414) = 37.11, p <.001		

Note. The covariables are described as Cov. 1 to Cov. 7 and are the following: Cov.1 is ‘Psycological Conditions’ (0 = Never had psychological support, 1= Already had psychological support sometime in my life; Cov.2 is ‘Caring for dependent people during quarantine’ (0 = No; 1= Yes); Cov. 3 is ‘Being in Quarantine’ (0 = No; 1= Yes); Cov. 4 is ‘Age’; Cov. 5 is ‘Gender’ (0 = Female; 1= Male); Cov. 6 is ‘Educational Level’ (1=Primary school to 7=Doctoral Studies); Cov. 7 is ‘Problematic/Important Physical Conditions’ (0 = No; 1= Yes).

Table S2

Unstandardized Regression Coefficients, Standard Errors, and Model Summary Information for the Parallel Multiple Mediator Model 2, considering Anxiety as the Dependent Variable.

	Dependent Variables																				
	Emotional Coping (M1)			Problem Oriented Coping (M2)			Dysfunctional Coping (M3)			Positive Affect (M4)			Negative Affect (M5)			Anxiety (Y)			Anxiety (Y)		
Predictors	Coeff.	*SE*	*p*	Coeff.	*SE*	*p*	Coeff.	*SE*	*p*	Coeff.	*SE*	*p*	Coeff.	*SE*	*p*	Coeff.	*SE*	*p*	Coeff.	*SE*	*p*
Self-Comp. (X)	0.14	0.01	0.00	0.07	0.01	0.00	-0.08	0.01	0.00	0.07	0.01	0.00	-0.11	0.01	0.00	-0.09	0.01	0.00	-0.04	0.01	0.00
M1	-	-	-	-	-	-	-	-	-	-	-	-	-	-	-	-	-	-	0.00	0.03	0.97
M2	-	-	-	-	-	-	-	-	-	-	-	-	-	-	-	-	-	-	0.01	0.05	0.8
M3	-	-	-	-	-	-	-	-	-	-	-	-	-	-	-	-	-	-	0.04	0.03	0.19
M4	-	-	-	-	-	-	-	-	-	-	-	-	-	-	-	-	-	-	-0.02	0.03	0.57
M5	-	-	-	-	-	-	-	-	-	-	-	-	-	-	-	-	-	-	0.43	0.03	0.00
Cov. 1	1.88	0.47	0.00	0.53	0.34	0.12	1.13	0.4	0.00	-0.33	0.41	0.42	0.57	0.4	0.15	1.05	0.31	0.00	0.74	0.26	0.00
Cov. 2	-0.85	0.46	0.07	-0.07	0.33	0.83	-0.15	0.39	0.70	-0.03	0.4	0.93	1.37	0.39	0.00	0.26	0.30	0.39	-0.32	0.25	0.2
Cov. 3	-0.09	0.46	0.84	-0.04	0.33	0.90	0.41	0.38	0.29	0.43	0.4	0.28	0.64	0.39	0.1	0.37	0.30	0.22	0.08	0.24	0.75
Cov. 4	0.01	0.02	0.66	-0.01	0.01	0.39	-0.02	0.02	0.32	0.00	0.02	0.94	-0.03	0.02	0.13	-0.03	0.01	0.02	-0.02	0.01	0.1
Cov. 5	-1.67	0.52	0.00	-0.92	0.37	0.01	-1.21	0.44	0.01	0.62	0.45	0.17	-1.74	0.44	0.00	-1.14	0.34	0.00	-0.31	0.29	0.28
Cov. 6	-0.01	0.17	0.93	0.21	0.12	0.08	-0.26	0.14	0.06	0.38	0.14	0.01	-0.09	0.14	0.54	-0.30	0.11	0.00	-0.25	0.09	0.01
Cov. 7	-0.35	0.53	0.5	0.02	0.38	0.96	-0.46	0.45	0.31	0.05	0.46	0.91	0.99	0.45	0.03	1.27	0.34	0.00	0.86	0.28	0.00
Constant	4.35	1.62	0.01	3.84	1.16	0.00	19.64	1.37	0.00	6.53	1.41	0.00	20.16	1.38	0.00	12.53	1.05	0.00	3.02	1.18	0.01
	R^2 = 0.23			R^2 = 0.15			R^2 = 0.19			R^2 = 0.11			R^2 = 0.27			R^2 = 0.32			R^2 = 0.56		
	F(8,419) = 15.79, p <.001			F(8,419) = 9.19, p <.001			F(8,419) = 12.17, p <.001			F(8,419) = 6.63, p <.001			F(8,419) = 19.60, p <.001			F(8,419) = 25.16, p <.001			F(13,414) = 39.87, p <.001		

Note. The covariables are described as Cov. 1 to Cov. 7 and are the following: Cov.1 is ‘Psycological Conditions’ (0 = Never had psychological support, 1= Already had psychological support sometime in my life; Cov.2 is ‘Caring for dependent people during quarantine’ (0 = No; 1= Yes); Cov. 3 is ‘Being in Quarantine’ (0 = No; 1= Yes); Cov. 4 is ‘Age’; Cov. 5 is ‘Gender’ (0 = Female; 1= Male); Cov. 6 is ‘Educational Level’ (1=Primary school to 7=Doctoral Studies); Cov. 7 is ‘Problematic/Important Physical Conditions’ (0 = No; 1= Yes).

Table S3

Unstandardized Regression Coefficients, Standard Errors, and Model Summary Information for the Parallel Multiple Mediator Model 3, considering Stress as the Dependent Variable.

	Dependent Variables																				
	Emotional Coping (M1)			Problem Oriented Coping (M2)			Dysfunctional Coping (M3)			Positive Affect (M4)			Negative Affect (M5)			Stress (Y)			Stress (Y)		
Predictors	Coeff.	*SE*	*p*	Coeff.	*SE*	*p*	Coeff.	*SE*	*p*	Coeff.	*SE*	*p*	Coeff.	*SE*	*p*	Coeff.	*SE*	*p*	Coeff.	*SE*	*p*
Self-Comp. (X)	0.14	0.01	0.00	0.07	0.01	0.00	-0.08	0.01	0.00	0.07	0.01	0.00	-0.11	0.01	0.00	-0.13	0.01	0.00	-0.08	0.01	0.00
M1	-	-	-	-	-	-	-	-	-	-	-	-	-	-	-	-	-	-	-0.05	0.04	0.23
M2	-	-	-	-	-	-	-	-	-	-	-	-	-	-	-	-	-	-	0.17	0.06	0.00
M3	-	-	-	-	-	-	-	-	-	-	-	-	-	-	-	-	-	-	0.08	0.04	0.07
M4	-	-	-	-	-	-	-	-	-	-	-	-	-	-	-	-	-	-	0.00	0.04	0.99
M5	-	-	-	-	-	-	-	-	-	-	-	-	-	-	-	-	-	-	0.48	0.04	0.00
Cov. 1	1.88	0.47	0.00	0.53	0.34	0.12	1.13	0.4	0.00	-0.33	0.41	0.42	0.57	0.4	0.15	1.95	0.38	0.00	1.59	0.32	0.00
Cov. 2	-0.85	0.46	0.07	-0.07	0.33	0.83	-0.15	0.39	0.70	-0.03	0.4	0.93	1.37	0.39	0.00	0.92	0.37	0.01	0.25	0.31	0.43
Cov. 3	-0.09	0.46	0.84	-0.04	0.33	0.90	0.41	0.38	0.29	0.43	0.4	0.28	0.64	0.39	0.1	0.63	0.37	0.09	0.29	0.31	0.34
Cov. 4	0.01	0.02	0.66	-0.01	0.01	0.39	-0.02	0.02	0.32	0.00	0.02	0.94	-0.03	0.02	0.13	-0.04	0.02	0.02	-0.02	0.01	0.12
Cov. 5	-1.67	0.52	0.00	-0.92	0.37	0.01	-1.21	0.44	0.01	0.62	0.45	0.17	-1.74	0.44	0.00	-1.83	0.42	0.00	-0.83	0.36	0.02
Cov. 6	-0.01	0.17	0.93	0.21	0.12	0.08	-0.26	0.14	0.06	0.38	0.14	0.01	-0.09	0.14	0.54	-0.09	0.13	0.5	-0.07	0.11	0.56
Cov. 7	-0.35	0.53	0.5	0.02	0.38	0.96	-0.46	0.45	0.31	0.05	0.46	0.91	0.99	0.45	0.03	0.83	0.43	0.05	0.37	0.36	0.3
Constant	4.35	1.62	0.01	3.84	1.16	0.00	19.64	1.37	0.00	6.53	1.41	0.00	20.16	1.38	0.00	18.16	1.31	0.00	6.55	1.49	0.00
	$R^2 = 0.23$			$R^2 = 0.15$			$R^2 = 0.19$			$R^2 = 0.11$			$R^2 = 0.27$			$R^2 = 0.40$			$R^2 = 0.59$		
	$F(8,419) = 15.79, p < .001$			$F(8,419) = 9.19, p < .001$			$F(8,419) = 12.17, p < .001$			$F(8,419) = 6.63, p < .001$			$F(8,419) = 19.60, p < .001$			$F(8,419) = 34.66, p < .001$			$F(13,414) = 46.01, p < .001$		

Note. The covariables are described as Cov. 1 to Cov. 7 and are the following: Cov.1 is ‘Psycological Conditions’ (0 = Never had psychological support, 1= Already had psychological support sometime in my life; Cov.2 is ‘Caring for dependent people during quarantine’ (0 = No; 1= Yes); Cov. 3 is ‘Being in Quarantine’ (0 = No; 1= Yes); Cov. 4 is ‘Age’; Cov. 5 is ‘Gender’ (0 = Female; 1= Male); Cov. 6 is ‘Educational Level’ (1=Primary school to 7=Doctoral Studies); Cov. 7 is ‘Problematic/Important Physical Conditions’ (0 = No; 1= Yes).

Table S4

Unstandardized Regression Coefficients, Standard Errors, and Model Summary Information for the Parallel Multiple Mediator Model 4, considering Stress as the Dependent Variable and the Problem Oriented Coping Strategies and Negative Affect as Mediators.

	Dependent Variables																	
	Active Coping (M1)			Planning (M2)			Use of instrumental support (M3)			Negative Affect (M4)			Stress (Y)			Stress (Y)		
Predictors	Coeff.	*SE*	*p*	Coeff.	*SE*	*p*	Coeff.	*SE*	*p*	Coeff.	*SE*	*p*	Coeff.	*SE*	*p*	Coeff.	*SE*	*p*
Self-Comp. (X)	0.04	0.00	0.00	0.02	0.00	0.00	0.02	0.01	0.00	-0.11	0.01	0.00	-0.13	0.01	0.00	-0.09	0.01	0.00
M1	-	-	-	-	-	-	-	-	-	-	-	-	-	-	-	-0.03	0.16	0.87
M2	-	-	-	-	-	-	-	-	-	-	-	-	-	-	-	0.47	0.15	0.00
M3	-	-	-	-	-	-	-	-	-	-	-	-	-	-	-	0.05	0.1	0.61
M4	-	-	-	-	-	-	-	-	-	-	-	-	-	-	-	0.49	0.04	0.00
Cov. 1	0.14	0.12	0.24	0.11	0.13	0.41	0.28	0.17	0.11	0.56	0.40	0.16	1.95	0.38	0.00	1.62	0.32	0.00
Cov. 2	-0.01	0.12	0.95	0.07	0.13	0.58	-0.14	0.17	0.42	1.38	0.39	0.00	0.92	0.37	0.01	0.21	0.31	0.51
Cov. 3	0.05	0.12	0.65	-0.01	0.13	0.91	-0.08	0.17	0.64	0.63	0.39	0.11	0.63	0.37	0.09	0.33	0.31	0.28
Cov. 4	0.00	0.01	0.65	0.00	0.01	0.94	-0.01	0.01	0.04	-0.03	0.02	0.13	-0.04	0.02	0.02	-0.02	0.01	0.08
Cov. 5	-0.18	0.14	0.19	-0.14	0.15	0.35	-0.61	0.19	0.00	-1.74	0.44	0.00	-1.83	0.42	0.00	-0.89	0.36	0.01
Cov. 6	0.10	0.04	0.03	0.09	0.05	0.06	0.03	0.06	0.68	-0.09	0.14	0.55	-0.09	0.13	0.5	-0.09	0.11	0.42
Cov. 7	0.04	0.14	0.75	-0.10	0.15	0.50	0.07	0.20	0.71	1.01	0.45	0.03	0.83	0.42	0.05	0.38	0.35	0.29
Constant	0.44	0.43	0.30	1.53	0.46	0.00	1.88	0.60	0.00	20.12	1.38	0.00	18.17	1.31	0.00	7.46	1.33	0.00
	$R^2 = 0.21$			$R^2 = 0.10$			$R^2 = 0.06$			$R^2 = 0.27$			$R^2 = 0.40$			$R^2 = 0.59$		
	$F(8,420) = 13.97, p < .001$			$F(8,420) = 5.92, p < .001$			$F(8,420) = 3.45, p = .001$			$F(8,420) = 19.55, p < .001$			$F(8,420) = 34.86, p < .001$			$F(12,416) = 50.24, p < .001$		

Note. The covariables are described as Cov. 1 to Cov. 7 and are the following: Cov.1 is ‘Psycological Conditions’ (0 = Never had psychological support, 1= Already had psychological support sometime in my life; Cov.2 is ‘Caring for dependent people during quarantine’ (0 = No; 1= Yes); Cov. 3 is ‘Being in Quarantine’ (0 = No; 1= Yes); Cov. 4 is ‘Age’; Cov. 5 is ‘Gender’ (0 = Female; 1= Male); Cov. 6 is ‘Educational Level’ (1=Primary school to 7=Doctoral Studies); Cov. 7 is ‘Problematic/Important Physical Conditions’ (0 = No; 1= Yes).
